# Comparison of nephroscopy and cystoscopy used in the treatment of bladder stones: a systematic review and meta-analysis of randomized controlled trials

**DOI:** 10.1186/s12893-021-01461-3

**Published:** 2021-12-31

**Authors:** Liping Gou, Zhenghao Wang, Ye Zhou, Xiaofeng Zheng

**Affiliations:** Department of Endocrinology and Metabolism, Center for Diabetes and Metabolism Research, West China Hospital, Sichuan University, Xinchuan Road 222, 610041 Chengdu, China

**Keywords:** BS, Transurethral, Endoscopic treatments, Cystoscope, Nephroscope

## Abstract

**Background:**

A systematic review and meta-analysis was conducted to compare the safety and efficiency of nephroscopy and cystoscopy in transurethral cystolithotripsy (TUCL) for bladder stones (BS).

**Methods:**

The PubMed, Web of Science, Embase, EBSCO, and Cochrane Library databases were searched up to January 2021 for studies assessing the effect of different types of endoscopes among patients who underwent TUCL. The search strategy and study selection process were in accordance with the PRISMA statement.

**Results:**

Five randomized controlled trials were included in the meta-analysis. The results showed no difference in stone-free rate (RR = 1.00, CI = 0.98–1.02, p = 1.00) between the two groups and nonsignificant heterogeneity (I^2^ = 0%, p = 1.00), and all patients were rendered stone free. Use of the nephroscope significantly shortened the operative time compared with the cystoscope group (RR= − 26.26, CI = − 35.84 to − 16.68, p < 0.00001), and there was significant heterogeneity (I^2^= 87%, p < 0.00001). There was no significant difference in mean urethral entries (RR = 0.66, CI = − 0.71 to − 2.04, p = 0.35), hospitalization (MD = 0.08, 95% CI = − 0.07 to 0.23, p = 0.31) or total complication rate (RR=1.37, 95% CI = 0.47–4.00, p = 0.56) between the two groups.

**Conclusions:**

In conclusion, this systematic review demonstrates that both nephroscopy and cystoscopy have high stone clearance efficiency, low rates of complications and short hospitalizations. The mean urethral entries depend on the treatment method for large stone fragments. However, the use of nephroscopy can significantly reduce the operative time.

## Background

Although improvements in modern antibiotic treatment and nutrition have decreased the incidence of bladder stones (BSs) in recent decades [1], BSs are still the most common type of stone in the lower urinary tract, accounting for 5% of all urinary calculi [2]. BSs are commonly classified as primary, secondary, and migratory [3], which are often associated with neurogenic voiding dysfunction, bladder outlet obstruction and urinary tract infection [4].

Compared to conservative therapy, such as medication therapy or chemolysis, surgical approaches are more efficient. Several surgical procedures for the treatment of BS have been developed, including open or transurethral surgery, percutaneous procedures, and extracorporeal shock wave lithotripsy (ESWL). Techniques for endoscopic surgery have also been developed, and traditional open cystolithotomy (OC) has been performed with minimally invasive treatments [5]. In addition to stone size, location and composition, factors including age, accompanying diseases and anatomy of the patients determine the choice of the surgical method. SWL is a simple, well tolerated and effective approach. However, the passage of residual fragments will be prolonged, and it is difficult to remove the large stones with this intervention alone [6]. Transurethral cystolithotripsy (TUCL) and percutaneous cystolithotripsy (PCCL) are considered to be the most frequently preferred approaches compared to others. Several lithotripsy energies, including holmium lasers and ultrasonic and pneumatic lithotriptres, are applied for the fragmentation of BSs [7]. Although there are a variety of approaches and modalities of lithotripsy, the treatment of BSs is still challenging. The complication rates and operative time change according to the treatment modality. Moreover, the type of endoscope also influences the safety and efficiency of the surgery. In general, transurethral surgery is normally performed by using a nephroscope or cystoscope [8].

The systematic review performed by Donaldson et al. compared different routines and energies in their studies and demonstrated that endoscopic, transurethral, and percutaneous BS treatments were associated with the same SFR (stone free rate) but reduced the duration of operation, hospitalization and catheterization compared with OC in patients of all ages. They also reported that mechanical, pneumatic, and laser lithotripsy exhibit the same efficiency for transurethral approaches [9]. However, only the stone-free rate (SFR) and operative time were compared between the nephroscope and cystoscope in their study. Furthermore, only one included study compared the percutaneous method with the transurethral method, and the number of included studies was limited [10]. Therefore, we conducted the first systematic review to compare the safety and efficiency of nephroscopy and cystoscopy in TUCL for BS.

## Materials and methods

This systematic review and meta-analysis followed the guidelines of the Preferred Reporting Items for Systematic Reviews and Meta-analysis (PRISMA) statement and the Cochrane Handbook for Systematic Reviews of Interventions [11]. Ethical approval and patient consent were not required because all analyses were based on previously published studies.

### Literature search and selection criteria

We systematically searched several databases, including PubMed, Embase, Web of Science, EBSCO, and the Cochrane Library, from inception to January 2021 with the following keywords: “bladder,” “calculus,” “cystoscope,” “nephroscope,” and “cystoscope.” The reference lists of retrieved studies and relevant reviews were manually searched, and the process mentioned above was repeatedly performed to ensure that all eligible studies were included.

The inclusion criteria were as follows: (1) the study design was a randomized controlled trial (RCT), (2) the patient had a history of bladder calculus and underwent transurethral cystolithotripsy, (3) the intervention approach was cystoscopy versus nephroscopy, and (4) the entire text was available. Studies reported in all languages were included. Study searching and data extraction were independently performed by two investigators (G.L. P and W.Z. H), and discrepancies were resolved by consensus. The primary outcomes were SFR and operative time, and the secondary outcomes were mean urethral entries, hospitalization and complications. The initial screening was performed by reading the titles and abstracts. All articles that potentially met the inclusion criteria were included. Then, duplicates were identified using EndNote (version x9).

### Quality assessment of individual studies

The methodological quality of each RCT was assessed according to the Jadad scale, which comprises the following three evaluation elements: randomization (0–2 points), blinding (0–2 points), and dropouts and withdrawals (0–1 points). One point was awarded for each element that was conducted and appropriately described in the original article. The total score ranged from 0 to 5 points. An article with a Jadad score of ≤ 2 was considered to be of low quality, while a Jadad score of ≥ 3 indicated a high-quality study [12].

## Results

### Literature search, study characteristics, and quality assessment

**Fig. 1:**
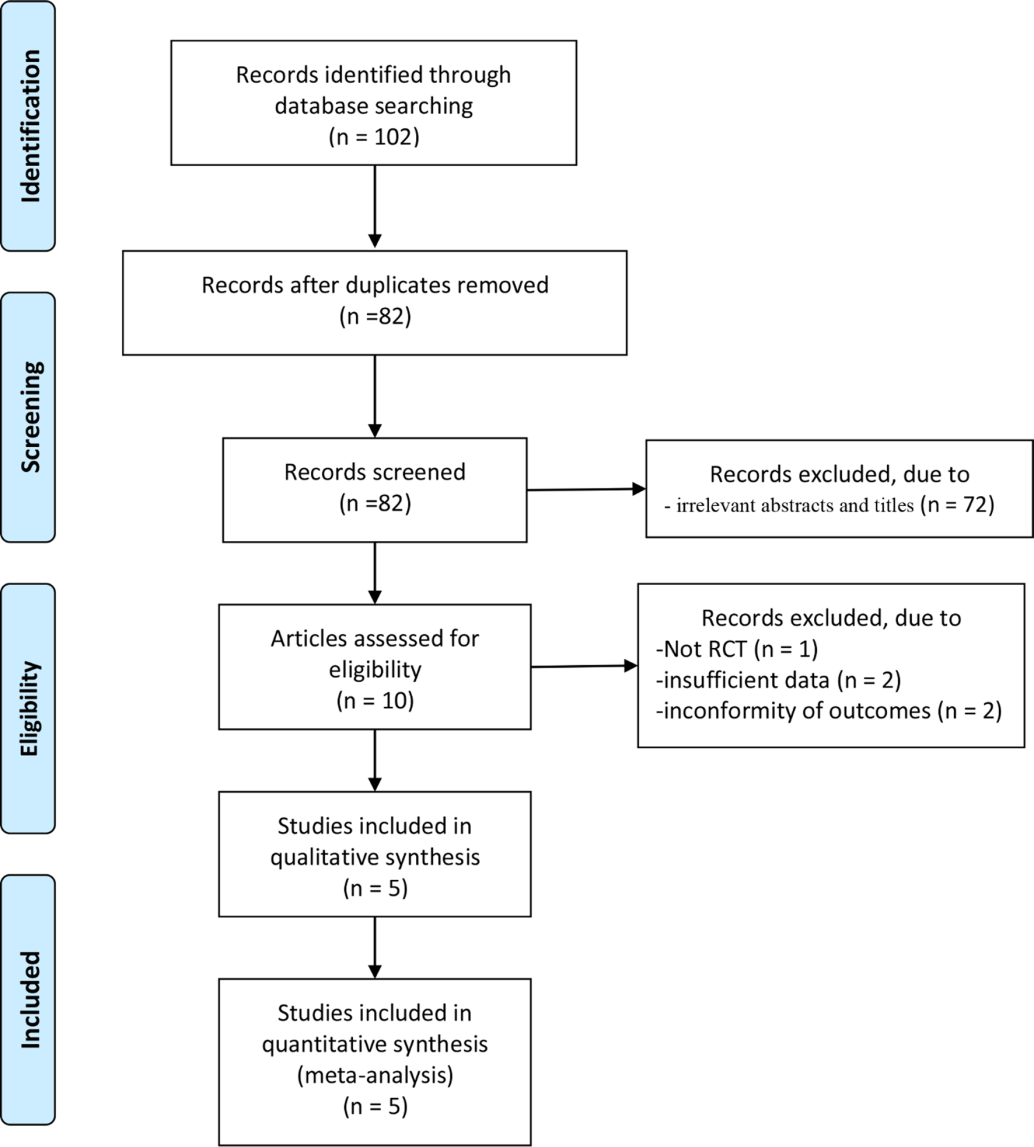
Flow diagram of the study search and selection process

In total, 102 articles were initially retrieved from the databases. After removing duplicates, 82 articles remained. Seventy-two studies were excluded from our study due to unrelated abstracts and titles. We also excluded 5 studies from our analysis: one for not having an RCT design, two for insufficient data, and two for not assessing the outcomes of interest. Finally, five RCTs satisfying the inclusion criteria were included in this meta-analysis [13–17]. The article selection process was performed in accordance with the PRISMA statement (Fig. 1). The baseline characteristics of the five included studies are shown in Table 1. Four studies took 24 Fr, and one study took 22 Fr in the nephroscope group. Four groups [13–15, 17] used 22 Fr, and one group used 23 Fr [16] in the cystoscope group. All studies used pneumatic lithotripsy with or without ultrasonic lithotripsy as the fragmentation energy. Singh et al. did not report the experience of surgeons [15]. The surgeries were conducted by the same experienced surgeon in four other studies. There were no statistically significant differences in baseline indicators. The studies in our meta-analysis were published between 2005 and 2020, and the total sample size was 791. The mean Jadad score ranged from 2 to 5. The main limitations of the included studies are a lack of blinding methods and descriptions of specific randomization methods [13, 16, 17]. The Jadad scores of each study are presented in Table 1.

**Table 1:** Characteristics of included studies

No.	Author	Year	Nephroscope group					Cystoscope group					Jada Score
			Number (n)	Age (Mean ± SD)	Male (n)	Stone size (cm)	Scope and energy	Number (n)	Age (Mean ± SD)	Male (n)	Stone size	Scope and energy	
1	Jang	2019	42	65.7 ± 13.5	37	5.24 ± 4.8	24Fr; ultrasonic or pneumatic lithotripsy	65	66.7 ± 13.5	57	5.55 ± 4.3	22 Fr; pneumatic lithotripsy	^2^
2	Bansal	2016	70	42.3 ± 21.6	61	2.9 ± 0.9	22 Fr; pneumatic lithotripsy	70	46.3 ± 23.7	64	2.6 ± 1.3	22 Fr; pneumatic lithotripsy	^5^
3	Ozdemir	2012	24	49.5 ± 9.50	24	4.34 ± 0.78	24Fr; combined pneumatic-ultrasonic lithotripsy	22	49.95 ± 11.38	24	4.28 ± 0.55	23Fr; combined pneumatic-ultrasonic lithotripsy	^2^
4	Singh	2011	20	45.6 ± 11.2	15	2.9 ± 1.1	24Fr; pneumatic lithotripsy	23	48.4 ± 12.3	16	3.1 ± 1.3	22 Fr; pneumatic lithotripsy	^3^
5	Ener	2009	22	48.6 ± 11.4	22	3.6 ± 1.3	24Fr; combined pneumatic-ultrasonic lithotripsy	21	49.9 ± 9.5	22	3.5 ± 1.6	22Fr; combined pneumatic-ultrasonic lithotripsy	^3^

### Primary outcomes

#### Stone clearance

**Fig. 2:**
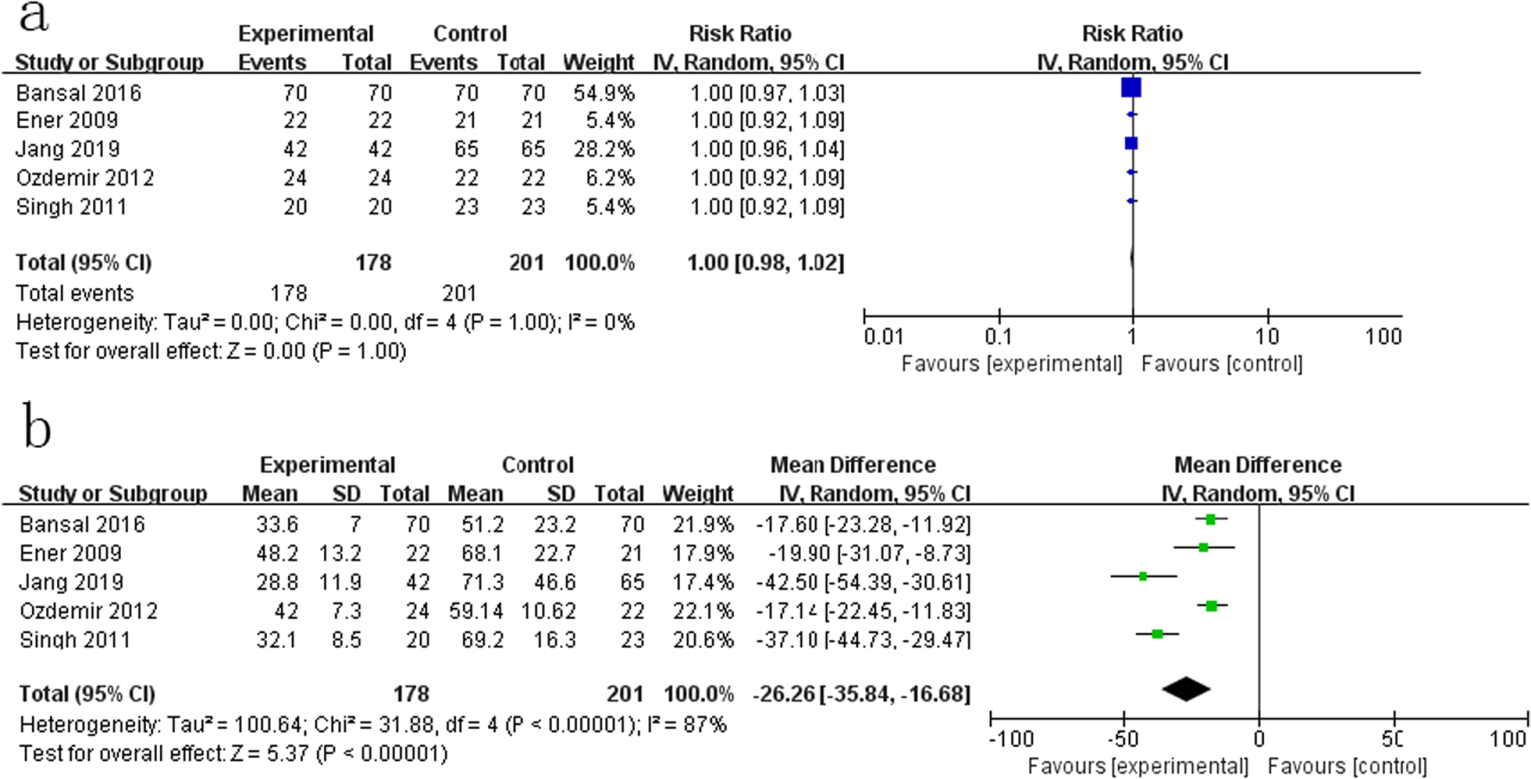
Forest plot for primary outcomes: **a** stone clearance. **b** Operation time

All studies reported the SFR. Three studies assessed the SFR by KUB or ultrasonography [15–17], and two studies [13, 14] did not mention it. The results showed no difference in SFR (RR = 1.00, CI = 0.98–1.02, p = 1.00, Fig. 2a) and nonsignificant heterogeneity (I^2^ = 0%, p =1.00), as all patients were rendered stone free.

#### Operative time

All studies reported the operative time. The meta-analysis revealed that the nephroscope group had a significantly shorter operative time than the cystoscope group (RR= − 26.26, CI = − 35.84 to − 16.68, p < 0.00001, Fig. 2b) with significant heterogeneity (I^2^ = 87%, p < 0.00001).

### Secondary outcome

#### Mean urethral entries

**Fig. 3:**
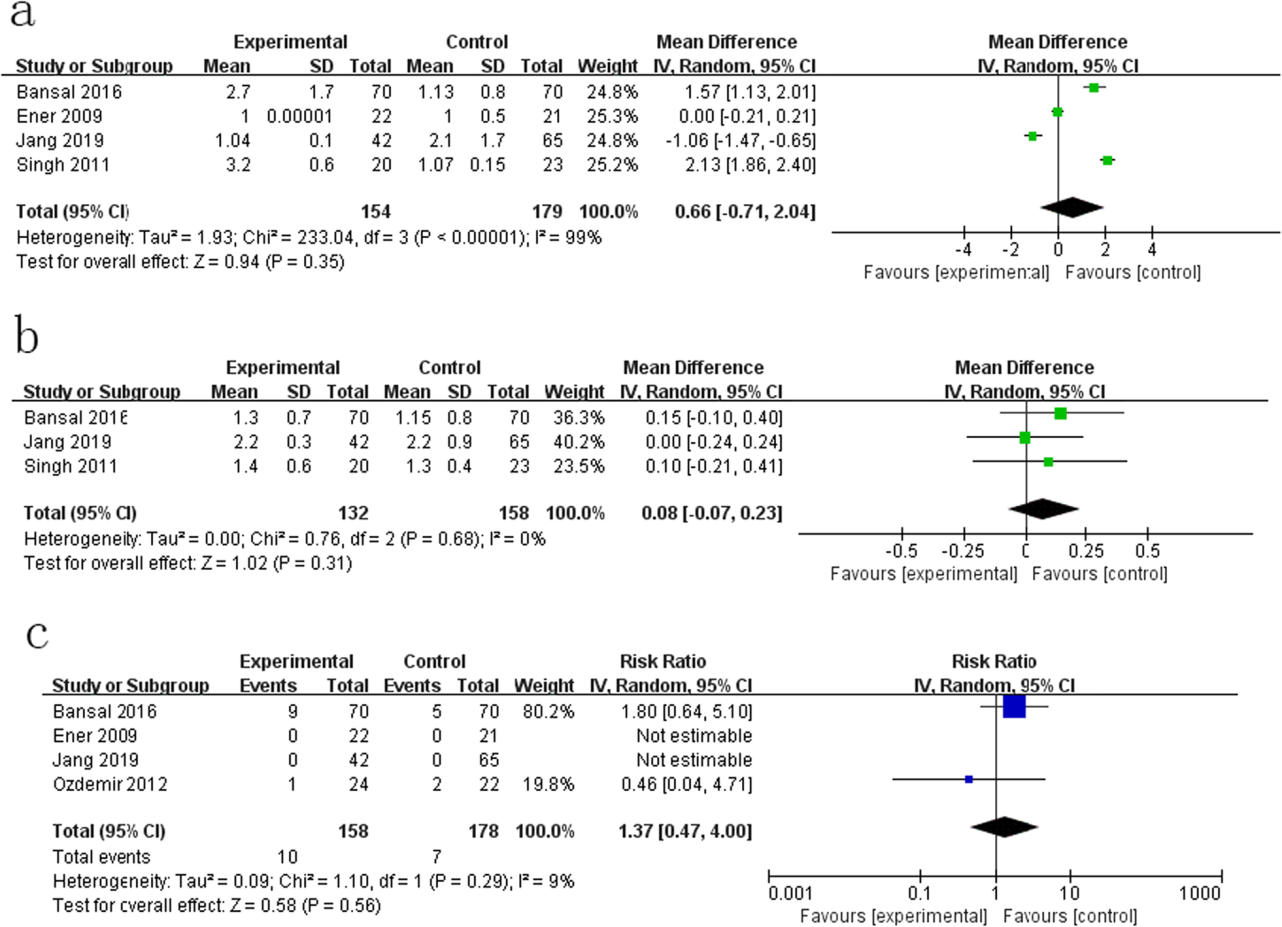
Forest plot for secondary outcomes: **a** mean urethral entries. **b** Hospitalization. **c** Total complications

Four studies reported the mean urethral entries [13–15, 17]. Our meta-analysis indicated that there was no difference in mean urethral entries between the cystoscope group and the nephroscope group (RR = 0.66; CI = − 0.71 to − 2.04, p = 0.35, Fig. 3a), with significant heterogeneity (I^2^ = 99%, p < 0.00001). Ozdemir et al. only reported the mean number of transurethral accesses and showed that nephroscopy (18.12 ± 4.94) can significantly reduce the number of transurethral accesses (26.81 ± 3.14) compared to cystoscopy (p <  0.0001) [16].

#### Hospitalization

Three studies [13–15] contained hospitalization data, and our results indicated that there was no significant difference between the two groups (MD = 0.08, 95% CI = − 0.07 to 0.23; p = 0.31), with nonsignificant heterogeneity (I^2^ = 0%, p = 0.63, Fig. 3b).

#### Total complication

Four studies [13, 14, 16, 17] reported the data of complications. No major complications were reported in these studies. Bansal et al. reported fever, transient haematuria, and urethral stricture in their study, and Ozdemir et al. [16] reported abrasion of the urethral mucosa with transient haematuria in their study. There was no difference between the two groups (RR = 1.37, 95% CI = 0.47–4.00, p = 0.56) with nonsignificant heterogeneity (I^2^ = 9%, p = 0.29, Fig. 3c).

#### Sensitivity analysis

Among all outcomes, operative time and mean urethral entries showed significant heterogeneity (I^2^ = 87%, p < 0.00001, I^2^ = 87%, p < 0.00001, respectively). Sensitivity analysis was performed to evaluate the stability of the results. After removing 1 study at a time, the heterogeneity values were I^2^ = 89%, 91%, 85%, 88% and 81% for operative time and I^2^ = 99%, 99%, 99% and 97% for mean urethral entries, indicating that the heterogeneities were stable.

## Discussion

A significant decrease in the incidence of BS and diversity of choice partly explain the relative paucity of contemporary scientific evidence for the best approach of treatment [18]. For the choice of the treatment of BS, many factors must be taken into consideration, including the composition and size of the stone, general condition of the patients, previous treatment history and anatomic abnormalities. Furthermore, the modalities of surgical equipment also play an important role in therapeutic success [19]. Transurethral surgery has become commonly used due to its high efficacy and low morbidity following the development of newer endoscopic and fragmentation equipment [20]. On the one hand, many lithotripsy energy facilities for stone crushing, such as electrohydraulic lithotripsy, ultrasonic lithotripsy, pneumatic lithotripsy and holmium:yttrium aluminium-garnet laser, are used via the transurethral approach, and their own weakness and merit have been well studied in previous studies [21–24]. On the other hand, diverse types of endoscopes, such as cystoscopes and nephroscopes, are also widely used in transurethral surgeries. However, whether one is better than the other in terms of operative characteristics and the incidence of complications remains unclear and controversial. Thus, we conducted the present systematic review to compare the effect and efficiency of these two types of endoscopes in procedures of removing BS.

Complete removal of stone fragments is the most important indicator for evaluating the efficacy of any stone surgery. The main result of our study indicates that there is no difference in SFR between the two endoscopes. All included patients reached a stone-free status by session despite the different endoscopes (cystoscope and nephroscope) and fragmentation equipment (ultrasonic, pneumatic or both). Donaldson et al. reported that the SFR of TUCL was the same as that of OC or PCCL but higher than that of ESWL in their systemic review [9]. Thus, we assume that the main influencing factor for the success rate is the TUCL method instead of devices. Another important evaluation criterion for stone treatment is operative time. Removing fragmented stones from the bladder is time-consuming, and operative time is the determining factor for relative complications and postoperative recovery in the TUCL procedure. Our results showed that the mean operative time for stone removal was significantly shorter in the nephroscope group. A slow inflow of saline and stopping intermittently can avoid overdistension of the bladder during surgery. When the bladder was distended, the bladder was emptied by removing rubber covering the port inlet of the nephroscope, providing a better view for the surgeon to evacuate stone fragments and save operative time [13]. Ener et al. assumed that the nephroscope has a larger lumen than the cystoscope, and it is better for removal of the calculus fragments through its lumen, which can also shorten the operative time [17].

There was no difference in terms of mean urethral entries. The heterogeneity of this outcome may be due to different methods of large fragment clearance. Bansal et al. used a cystoscope for the removal of fragments after removing the nephroscope, as they used an evacuator to rush out large fragments, which is only suitable for cystoscopy. After that, the nephroscope was inserted again for further fragmentation and retrieval of fragments [14]. For this reason, the mean urethral entries were much higher in the nephroscope group. Ener et al. and Ozdemir et al. kept the sheath in the urethra, preventing the need for multiple entries to the bladder during surgery while using a nephroscope, which is an effective and reliable method. Nevertheless, the removal of larger stone fragments necessitates pulling the cystoscope out with the stone together [16, 17]. Furthermore, Jang et al. also found that the larger diameter of the nephroscope is easier for removal of fragmented stones, which can significantly reduce the reinsertion of the scope [13]. There were no differences in hospitalization or total complication rates, which indicates that both scopes are safe and that no severe complications were reported in any patients. This can also be attributed to the minimum injury of the TUCL. Although the open and percutaneous approach offered a better view, prolonged instrumentation of the urethra was avoided. However, the longer the length of postoperative recovery and higher incidence of complications occurred due to the trauma and placement of the suprapubic catheter [14]. It is worth noting that the urethral stricture was higher (5/70 vs. 2/70) in the nephroscope group in the study of mucosal injury due to higher entries of the nephroscope [14].

Admittedly, there were several limitations to this study. First, the limitation of the included studies and patients is the major deficiency of this study. Second, our findings may be skewed because of some unpublished data and missing negative data in the original studies. Last, there were differences between the fragmentation equipment and diameter of the scopes that made assessing the efficacy of surgery. Lastly, a high-level prospective RCT with a large sample and a more consistent baseline should be conducted in the future. Furthermore, choosing surgical equipment for BS based only on its characteristics is very unrealistic in clinical practice. BS patients often with bladder outlet obstruction, such as BPH or bladder neck sclerosis, will also be a factor for surgeons to choose the instrument. Thus, we suppose that the choice of surgical equipment is based on a combination of all factors for urologists in daily work.

In conclusion, this systematic review demonstrates that both nephroscopy and cystoscopy have a high efficiency of stone clearance, a low rate of complications and short hospitalization. The mean urethral entries depend on the treatment method for large stone fragments. However, the use of nephroscopy can significantly reduce the operative time.

## Data Availability

The datasets used and/or analyzed during the current study are available from the corresponding author on reasonable request.

## References

[1] Wagner CA (2021). Etiopathogenic factors of urolithiasis. Arch Espanol Urol.

[2] Schwartz BF, Stoller ML (2000). The vesical calculus. Urol Clin North Am.

[3] Philippou P, Moraitis K, Masood J, Junaid I, Buchholz N (2012). The management of bladder lithiasis in the modern era of endourology. Urology.

[4] Yoshida O, Okada Y (1990). Epidemiology of urolithiasis in Japan: a chronological and geographical study. Urol Int.

[5] Torricelli FC, Mazzucchi E, Danilovic A, Coelho RF, Srougi M (2013). Surgical management of bladder stones: literature review. Rev Colegio Brasileiro de Cirurgioes.

[6] Papatsoris AG, Varkarakis I, Dellis A, Deliveliotis C (2006). Bladder lithiasis: from open surgery to lithotripsy. Urol Res.

[7] Razvi HA, Song TY, Denstedt JD (1996). Management of vesical calculi: comparison of lithotripsy devices. J Endourol.

[8] Toktas G, Sacak V, Erkan E, Kocaaslan R, Demiray M, Unluer E, Ozyalvacli ME (2013). Novel technique of cytolithotripsy for large bladder stones. Asian J Endosc Surg.

[9] Donaldson JF, Ruhayel Y, Skolarikos A, MacLennan S, Yuan Y, Shepherd R, Thomas K, Seitz C, Petrik A, Türk C (2019). Treatment of bladder stones in adults and children: a systematic review and meta-analysis on behalf of the European Association of Urology Urolithiasis Guideline Panel. Eur Urol.

[10] Zhao J, Shi L, Gao Z, Liu Q, Wang K, Zhang P (2013). Minimally invasive surgery for patients with bulky bladder stones and large benign prostatic hyperplasia simultaneously: a novel design. Urol Int.

[11] Liberati A, Altman DG, Tetzlaff J, Mulrow C, Gøtzsche PC, Ioannidis JP, Clarke M, Devereaux PJ, Kleijnen J, Moher D (2009). The PRISMA statement for reporting systematic reviews and meta-analyses of studies that evaluate health care interventions: explanation and elaboration. PLoS Med.

[12] Jadad AR, Moore RA, Carroll D, Jenkinson C, Reynolds DJ, Gavaghan DJ, McQuay HJ (1996). Assessing the quality of reports of randomized clinical trials: is blinding necessary?. Control Clin Trials.

[13] Jang JY, Ko YH, Song PH, Choi JY (2019). Comparison of three different endoscopic approaches in the treatment of bladder calculi. Yeungnam Univ J Med.

[14] Bansal A, Kumar M, Sankhwar S, Goel S, Patodia M, Aeron R, Bhaskar V (2016). Prospective randomized comparison of three endoscopic modalities used in treatment of bladder stones. Urologia.

[15] Singh KJ, Kaur J (2011). Comparison of three different endoscopic techniques in management of bladder calculi. Indian J Urol IJU J Urolog Soc India.

[16] TunçOzdemir A, Koyuncu H, Altinova S, Asil E, Isgoren E, Gurdal M (2014). Comparison of transurethral use of nephroscope with cystoscope in transurethral cystolithotripsy. J Clin Anal Med.

[17] Ener K, Agras K, Aldemir M, Okulu E, Kayigil O (2009). The randomized comparison of two different endoscopic techniques in the management of large bladder stones: transurethral use of nephroscope or cystoscope?. J Endourol.

[18] Javali T, Nayak KA, Babu S (2018). Simultaneous antegrade and retrograde endoscopic surgery for benign prostatic hyperplasia with vesical calculi—a single-centre experience. Arab J Urol.

[19] Sarkis J, Alkassis M, Chebel JA, Tabcheh A, Semaan A (2020). Bladder stone following intravesical migration of surgical clip five years after radical prostatectomy. Urology case reports.

[20] Roslan M, Przudzik M, Borowik M (2019). Endoscopic intact removal of medium-size- or multiple bladder stones with the use of transvesical laparoendoscopic single-site surgery. World J Urol.

[21] Bülow H, Frohmüller HG (1981). Electrohydraulic lithotripsy with aspiration of the fragments under vision—304 consecutive cases. J Urol.

[22] Maeda O, Usami M. Summary of ‘General Rule for Clinical and Pathological Studies on Prostate Cancer (the 3rd Edition) ’. Nihon Rinsho Japan J Clin Med. 2005;63(2):201–206. 15714966

[23] Bhatia V, Biyani CS (1994). A comparative study of cystolithotripsy and extracorporeal shock wave therapy for bladder stones. Int Urol Nephrol.

[24] Matsuoka K, Iida S, Nakanami M, Koga H, Shimada A, Mihara T, Noda S (1995). Holmium: yttrium-aluminum-garnet laser for endoscopic lithotripsy. Urology.

